# Incomplete Hydrogenation by Geranylgeranyl Reductase from a Proteobacterial Phototroph Halorhodospira halochloris, Resulting in the Production of Bacteriochlorophyll with a Tetrahydrogeranylgeranyl Tail

**DOI:** 10.1128/jb.00605-21

**Published:** 2022-03-15

**Authors:** Yusuke Tsukatani, Jiro Harada, Kanako Kurosawa, Keiko Tanaka, Hitoshi Tamiaki

**Affiliations:** a Institute for Extra-Cutting-Edge Science and Technology Avant-Garde Research (X-star), Japan Agency for Marine-Earth Science and Technology (JAMSTEC), Kanagawa, Japan; b Department of Medical Biochemistry, Kurume University School of Medicine, Fukuoka, Japan; c Graduate School of Life Sciences, Ritsumeikan University, Shiga, Japan; Queen Mary University of London

**Keywords:** anoxygenic photosynthetic bacteria, bacteriochlorophyll, geranylgeranyl reductase, isoprenoid, pigment biosynthesis, *Chlorobaculum tepidum*, *Halorhodospira halochloris*, chlorophyll, photosynthesis, purple bacteria

## Abstract

Light harvesting and charge separation are functions of chlorophyll and bacteriochlorophyll pigments. While most photosynthetic organisms use (bacterio)chlorophylls with a phytyl (2-phytenyl) group as the hydrophobic isoprenoid tail, Halorhodospira halochloris, an anoxygenic photosynthetic bacterium belonging to Gammaproteobacteria, produces bacteriochlorophylls with a unique 6,7,14,15-tetrahydrogeranylgeranyl (2,10-phytadienyl) tail. Geranylgeranyl reductase (GGR), encoded by the *bchP* gene, catalyzes hydrogenation at three unsaturated C=C bonds of a geranylgeranyl group, giving rise to the phytyl tail. In this study, we discovered that *H. halochloris* GGR exhibits only partial hydrogenation activities, resulting in the tetrahydrogeranylgeranyl tail formation. We hypothesized that the hydrogenation activity of *H. halochloris* GGR differed from that of Chlorobaculum tepidum GGR, which also produces a pigment with partially reduced hydrophobic tails (2,6-phytadienylated chlorophyll *a*). An engineered GGR was also constructed and demonstrated to perform only single hydrogenation, resulting in the dihydrogeranylgeranyl tail formation. *H. halochloris* original and variant GGRs shed light on GGR catalytic mechanisms and offer prospective bioengineering tools in the microbial production of isoprenoid compounds.

**IMPORTANCE** Geranylgeranyl reductase (GGR) catalyzes the hydrogenation of carbon–carbon double bonds of unsaturated hydrocarbons of isoprenoid compounds, including α-tocopherols, phylloquinone, archaeal cell membranes, and (bacterio)chlorophyll pigments in various organisms. GGRs in photosynthetic organisms, including anoxygenic phototrophic bacteria, cyanobacteria, and plants perform successive triple hydrogenation to produce chlorophylls and bacteriochlorophylls with a phytyl chain. Here, we demonstrated that the GGR of a gammaproteobacterium Halorhodospira halochloris catalyzed unique double hydrogenation to produce bacteriochlorophylls with a tetrahydrogeranylgeranyl tail. We also constructed a variant enzyme derived from *H. halochloris* GGR that performs only single hydrogenation. The results of this study provide new insights into catalytic mechanisms of multiposition reductions by a single enzyme.

## INTRODUCTION

Chlorophyll (Chl) and bacteriochlorophyll (BChl) pigments are critical in photosynthetic organisms for harvesting light energy and transferring it to photochemical reaction center (RC) complexes, where the charge separation takes place. Chl occurs in all oxygenic phototrophs (including plants, algae, and cyanobacteria) and some species of anoxygenic phototrophic bacteria with type-I RCs (1–3). In the green sulfur bacterium Chlorobaculum tepidum, Chl is attached to type-I RCs and functions as the primary electron acceptor A_0_, although BChls are major pigments in the bacterium (2). BChl pigments are detected in all species of anoxygenic phototrophic bacteria, regardless of whether they have type-I or type-II RCs, but not in oxygenic phototrophs.

Photosynthetic organisms biosynthesize pigments through a series of catalytic reactions by various enzymes (4, 5). Chl and BChl share early biosynthetic steps, from an initial substance 5-aminolevulinic acid to chlorophyllide *a* or divinyl chlorophyllide *a* (4–7). The committed biosynthetic step for BChl *a* is branched at the chlorophyllide *a* reduction stage (6), whereas the committed step for BChl *b* and BChl *g* is branched at the 8-vinyl chlorophyllide *a* (7–9). These two steps correspond to the conversion of a chlorin ring into a bacteriochlorin ring.

**FIG 1:**
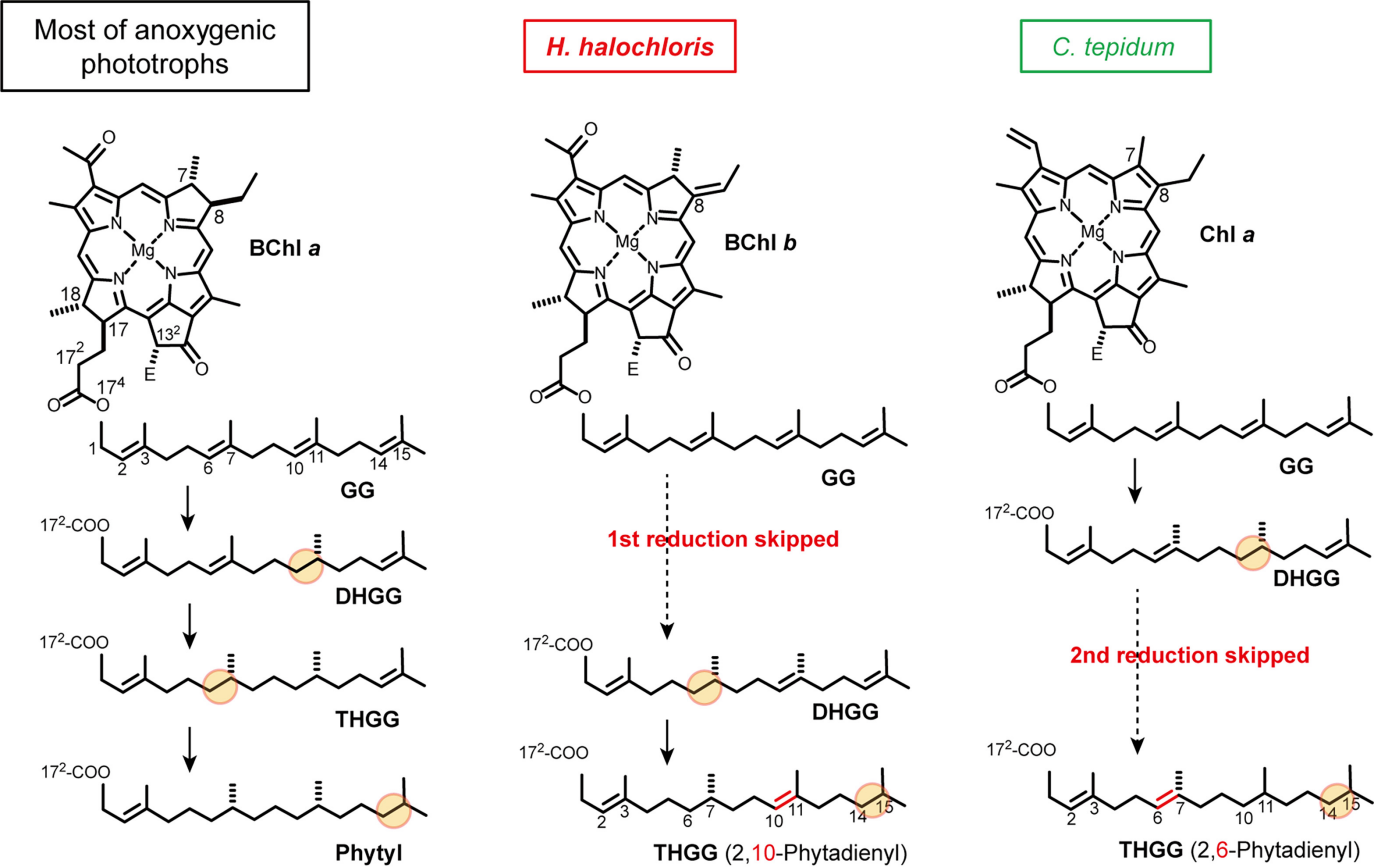
Reaction schemes for hydrogenation processes from the GG tail in (B)Chl biosynthesis. (Left panel) Proposed hydrogenation steps for phytyl formation in most phototrophic bacteria producing BChl *a*_P_. (Middle panel) Proposed hydrogenation steps for THGG formation in *H. halochloris* producing BChl *b*_THGG_. Note that BChl *a* has an ethyl group at the C8 position, whereas BChl *b* has a C8 ethylidene group. (Right panel) Proposed hydrogenation steps for THGG formation in *C. tepidum*, which produces Chl *a*_THGG_. “E” at the C13^2^ position represents COOCH_3_.

Although Chl and BChl species possess specific core π-skeletons and peripheral substituents, the penultimate and last biosynthetic steps for all the photosynthetic (B)Chl pigments except Chl c are common, where the order of the two steps can be switched. The esterification of geranylgeranyl diphosphate into the substituent at the carbon 17 (C17) position of (bacterio)chlorophyllide (10–12) is the penultimate step. The esterification is catalyzed by an enzyme designated as (B)Chl synthase encoded by the *bchG/chlG* gene. The last step is the hydrogenation of the C17 geranylgeranyl to the phytyl tail, which is catalyzed by *bchP/chlP* gene-encoded geranylgeranyl reductase (GGR) (10, 13). As depicted in Fig. 1, the geranylgeranyl tail is reduced three times to a phytyl group. The triple double-bond reduction of geranylgeranyl group by GGR occurred in the order of C10=C11, C6=C7, and C14=15 (Fig. 1, left column) (14).

Halorhodospira halochloris, a halophilic anoxygenic phototrophic bacterium belonging to the phylum Gammaproteobacteria, produces BChl *b* esterified with a unique isoprenoid tail at the C17 position, namely, a tetrahydrogeranylgeranyl (THGG) tail (Fig. 1, middle column) (15, 16). The THGG tail in *H. halochloris* is characterized as the C10=C11 unreduced double bond (thereby also called 2,10-phytadienyl) (15, 16), indicating that the first double-bond reduction of the triple hydrogenation occurring in other phototrophic bacteria is likely skipped or inhibited *in vivo* in *H. halochloris* (Fig. 1, middle). Therefore, the following two hypotheses emerge: (i) GGR in *H. halochloris* only reduces the isoprenoid tail twice, or (ii) GGR in *H. halochloris* potentially reduces three times, as observed in other phototrophic bacteria, but is prevented from reducing the C10=C11 double bond by an unidentified component(s). The latter case has been proposed for Chl biosynthesis in green sulfur bacteria (17, 18).

Green sulfur bacteria produce BChl *a* with the usual phytyl tail, but they also produce Chl *a* esterified with a unique THGG group (Fig. 1, right column) (2, 19). The hydrophobic THGG tail of Chl *a* in *C. tepidum* displays the C6=C7 unreduced double bond (2), which is different from the C10=C11 unreduced double bond in *H. halochloris* (Fig. 1). In the case of *C. tepidum*, a mutant lacking GGR accumulated BChl *a* and Chl *a* with the geranylgeranyl tails at the C17 position (17, 18), and it was concluded that a single *bchP* gene, *CT2256*, is responsible for saturating the geranylgeranyl tails esterified with both BChl *a* and Chl *a* in *C. tepidum*. Therefore, GGR (gene product of *CT2256*) of *C. tepidum* potentially has a catalytic ability of three reductions of the geranylgeranyl moiety, yielding the phytyl tail; however, this ability is somehow inhibited or unachieved in Chl biosynthesis but not in BChl biosynthesis. This *C. tepidum* GGR model correlates with the aforementioned second hypothesis. Recently, through an analysis of whole-genome sequencing of *H. halochloris*, a *bchP* gene was identified in the photosynthetic gene cluster of its genome (20). In this study, we investigated the catalytic activities of the *H. halochloris* GGR by creating a series of complementation mutants.

## RESULTS

**FIG 2:**
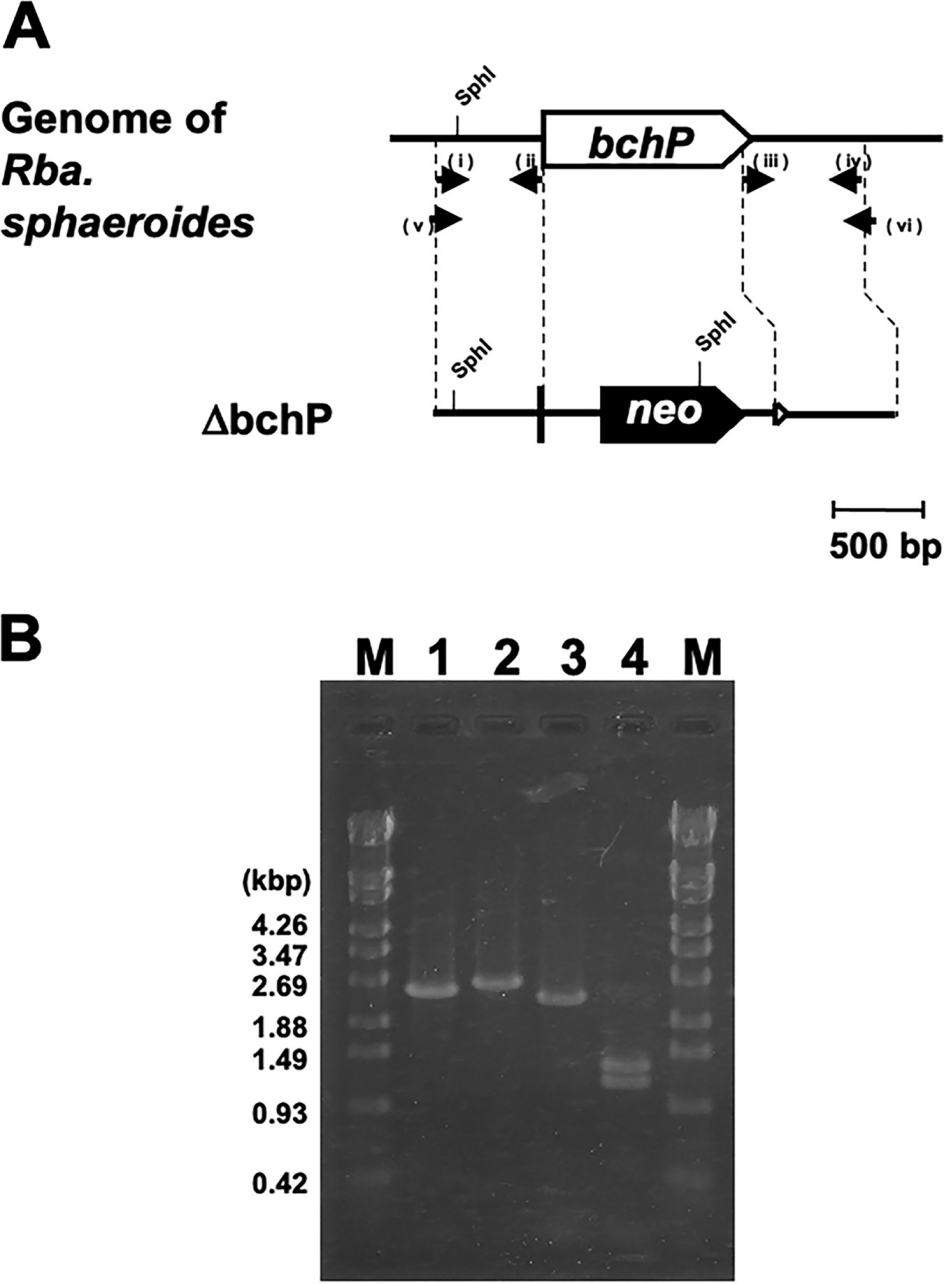
Construction of Δ*bchP* mutant as the host for complementation experiments. (A) Schematic drawing of the *bchP* locus of R. sphaeroides. The *bchP* gene was inactivated by replacement with the *neo* gene conferring resistance to kanamycin. Arrows represent the primers that were used in this study: (i) bchPus-F, (ii) bchPus-R, (iii) bchPud-F, (iv) bchPud-R, (v) sphaP-comf-F, and (vi) sphaP-comf-R. (B) Analytical PCR of the *bchP* locus of the wild-type and mutant R. sphaeroides. Using sphaP-comf-F and -R primers, the amplified PCR products from wild-type and *bchP*-deletion mutant strains are estimated to be 2.48 and 2.66 kbp, and were loaded onto lanes 1 and 2, respectively. Because of the length differences between the two PCR fragments, these were then digested by the restriction enzyme, SphI. The length of the digested PCR products from the wild type remained nearly unchanged (approximately 0.15 and 2.33 kbp, lane 3), whereas the PCR fragments from the *bchP* mutant were digested into three pieces (approximately 0.15, 1.17, and 1.34 kbp, lane 4). The DNA molecular weight marker, λ-EcoT14I digest (TaKaRa-Bio), was used to estimate the molecular mass of PCR products in lane M.

**FIG 3:**
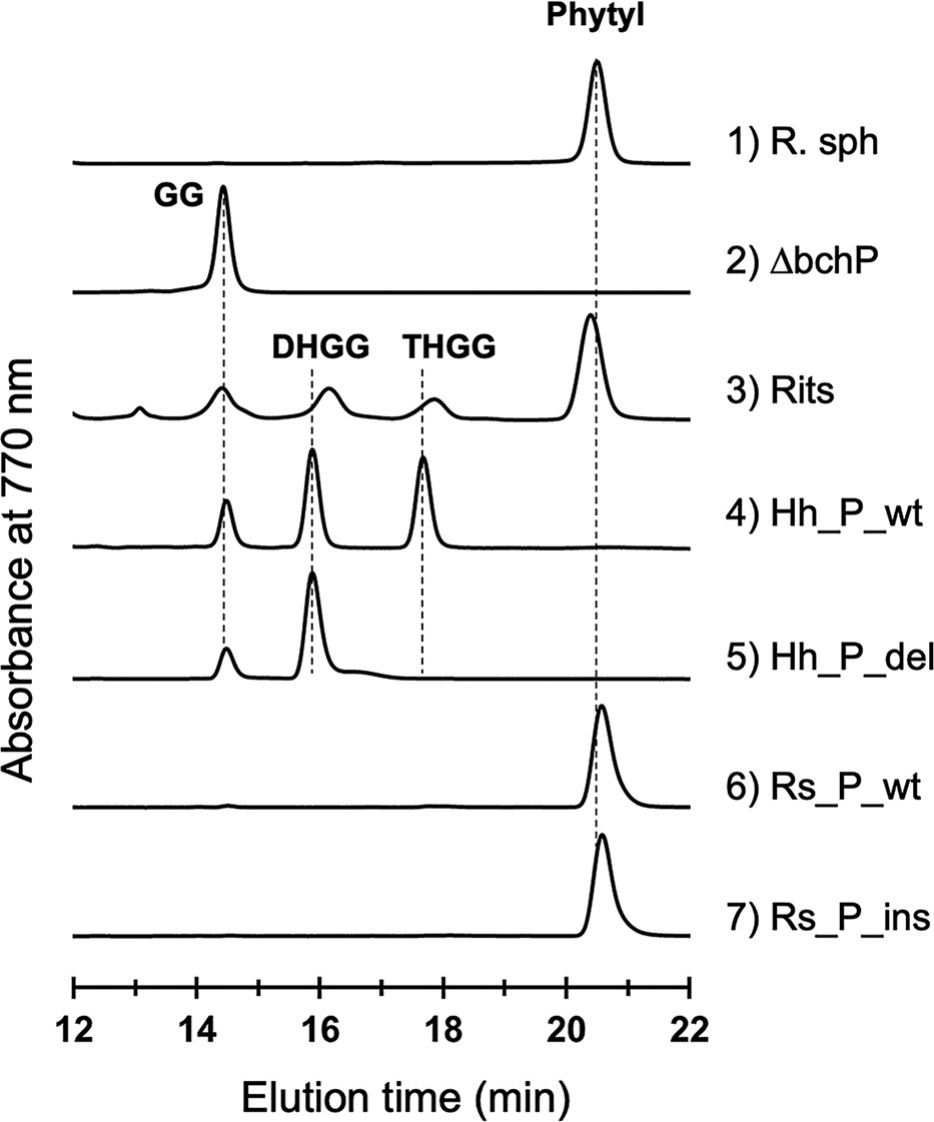
Reverse-phase HPLC elution profiles of pigment extracts from the wild-type and mutant strains. Pigments were extracted from 1) R. sphaeroides J001, 2) Δ*bchP* mutant, 3) *Rhodopseudomonas* sp. strain Rits, 4) Hh_P_wt, 5) Hh_P_del, 6) Rs_P_wt, and 7) Rs_P_ins.

We first constructed the Δ*bchP* mutant of R. sphaeroides lacking GGR (Fig. 2) (for details, see Materials and Methods). The Δ*bchP* mutant strain served as a host to construct complementation mutants containing wild-type and variant GGRs of H. halochloris and R. sphaeroides. We examined the pigment compositions of these mutant strains using high-performance liquid chromatography (HPLC) (Fig. 3). The HPLC elution profile of pigment extracts from the wild type of R. sphaeroides revealed an authentic phytylated BChl *a*, which was eluted at roughly 20.5 min (Fig. 3, profile 1). The Δ*bchP* mutant of R. sphaeroides lacking GGR did not exhibit the phytylated BChl *a* profile at 20.5 min; instead, the mutant accumulated geranylgeranylated BChl *a*, which was eluted at roughly 14.5 min (Fig. 3, profile 2). Profile 3 in Fig. 3 depicts the HPLC elution profile of pigment extracts from *Rhodopseudomonas* sp. strain Rits, which was discovered to accumulate BChl *a* molecules with unreduced and partially-reduced isoprenoid tails (14, 21). *Rhodopseudomonas* sp. strain Rits exhibited four elution peaks at 14.5, 16, 18, and 20.5 min, which were attributed to BChl *a* esterified with GG, dihydrogeranylgeranyl (DHGG), THGG, and phytyl tails, respectively, according to the previous study (21) (Fig. 3, profile 3). The HPLC elution profile of pigments extracted from the Hh_P_wt mutant, which has an intact GGR of *H. halochloris* in the background of the R. sphaeroides Δ*bchP* strain, revealed three peaks of BChl *a* esterified with GG, DHGG, and THGG tails (Fig. 3, profile 4), but no phytylated BChl *a*. The result indicates that the GGR of *H. halochloris* catalyzes only two double-bonds hydrogenation, and thereby the final product has the THGG tail, rather than the phytyl tail. Although both organisms produce (B)Chl pigments with THGG tails, the potential catalytic activity of *H. halochloris* GGR differs from that of *C. tepidum* GGR (17, 18). *C. tepidum* produces Chl *a* with the THGG tail, but Harada et al. showed that a mutant complemented with *C. tepidum* GGR produced phytylated BChl *a*, indicating that *C. tepidum* GGR can reduce a GG tail attached to a bacteriochlorin ring to a phytyl tail (17).

**FIG 4:**
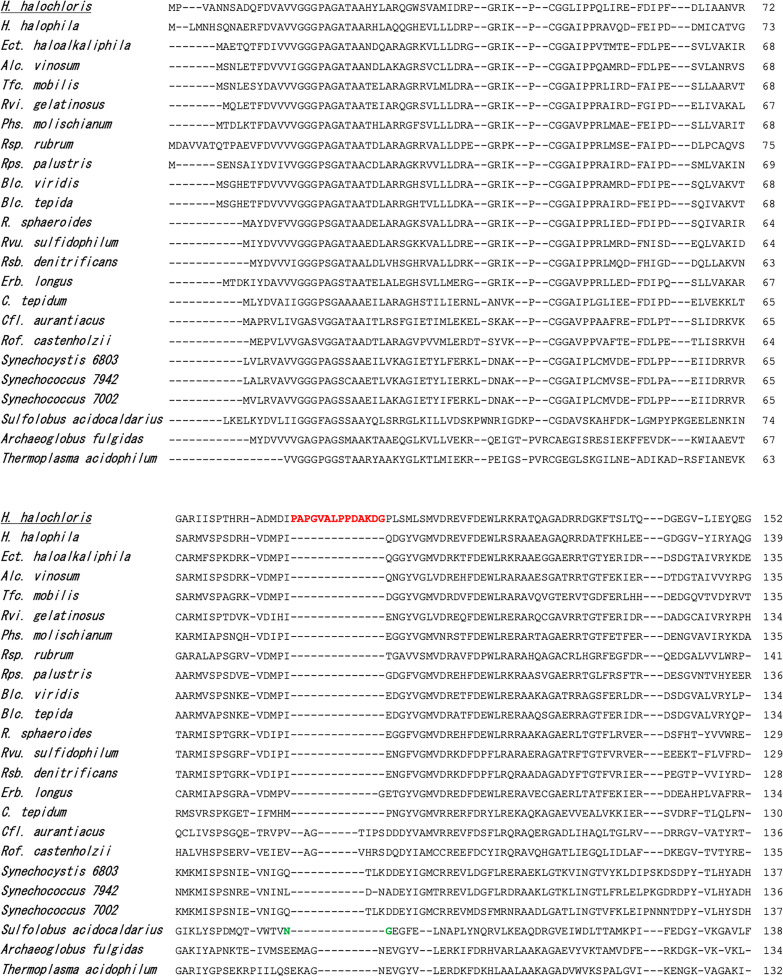
Partial alignments of the N-terminal region of GGR from various bacteria and archaea, including phototrophic members of Proteobacteria, Chlorobi, and Chloroflexi. The alignment was constructed using the MAFFT program (30) implemented in the Geneious Prime software (31). The characteristic insertion region shown in GGR (BchP) of *H. halochloris* is colored in red. Important residues close to the substrate-binding site in Sulfolobus acidocaldarius GGR crystal structure studies are colored in green. *H*., *Halorhodospira*; *Ect*., *Ectothiorhodospira*; *Rvi*., *Rubrivivax*; *Blc*., *Blastochloris*; *Alc*., *Allochromatium*; *Phs*., *Phaeospirillum*; *Rps*., *Rhodopseudomonas*; *Tfc*., *Thioflavicoccus*; *Erb*., *Erythrobacter*; *R*., *Rhodobacter*; *Rvu*., *Rhodovulum*; *Rsb*., *Roseobacter*; *Rsp*., *Rhodospirillum*; *C*., *Chlorobaculum*; *Cfl*., *Chloroflexus*; *Rof*., *Roseiflexus*.

Figure 4 depicts the N-terminus of the amino acid sequence alignment of GGR from several phototrophic bacteria and nonphotosynthetic archaea. *H. halochloris* GGR has a characteristic insertion at the N-terminal side of its primary sequence (Fig. 4, colored in red). We constructed the Δ*bchP* mutant of R. sphaeroides complemented with the variant GGR of *H. halochloris* that lacks the insertion region, designated Hh_P_del, because the insertion region could be relevant to presumably inhibiting the unachieved double-bond reduction at the C10=C11 position. The HPLC elution profile of pigments from the Hh_P_del mutant exhibited the GG peak eluting at roughly 14.5 min and the DHGG peak at around 16 min; however, the mutant did not accumulate peaks derived from BChl *a* with THGG and phytyl tails (Fig. 3, profile 5). The result indicates that the loss of the insertion region of *H. halochloris* GGR caused the loss of either the first or last hydrogenation and that the variant GGR catalyzes only a single hydrogenation reaction, yielding the DHGG tail.

We constructed a positive-control complementation mutation by introducing the intact *bchP* gene for R. sphaeroides GGR in the Δ*bchP* mutant of R. sphaeroides (designated as Rs_P_wt). The positive-control mutant restored the production of phytylated BChl *a*, as the HPLC elution peak of the pigment was observed at 20.5 min (Fig. 3, profile 6).

In a converse experiment to the Hh_P_del mutant, we constructed a mutant with a modified *bchP* gene, in which the characteristic insertion sequences encoding PAPGVALPPDAKDG (Fig. 4, colored in red) derived from *H. halochloris* were inserted into the corresponding region of R. sphaeroides *bchP* gene. The mutant designated Rs_P_ins accumulated phytylated BChl *a* (Fig. 3, profile 7), which was consistent with the result observed for the Rs_P_wt strain.

## DISCUSSION

It has been unknown whether a single GGR enzyme (BchP) in *H. halochloris* is responsible for the unusual THGG tail formation, or whether *H. halochloris* GGR can catalyze phytyl formation as observed in most phototrophs, but unknown protein(s) are involved in inhibiting the C10=C11 reduction in this bacterium. Here, we demonstrated that the heterologous expression of *H. halochloris* GGR in the R. sphaeroides mutant lacking its original GGR resulted in the accumulation of BChl *a* esterified with a THGG group. The results indicate that the GGR of *H. halochloris* itself is responsible for the production of the THGG moiety and that the enzymatic activity of GGR in the bacterium is distinct from that in most other phototrophic bacteria producing phytylated BChl *a*.

*C. tepidum*, a green sulfur bacterium, produces Chl *a* esterified with the THGG moiety, although it also biosynthesized BChl *a* with a regular phytyl tail. The THGG moiety detected in *C. tepidum* is 2,6-phytadienyl and therefore differs from 2,10-phytadienyl in *H. halochloris* (for structural comparison, see Fig. 1). Before recent studies, it had been assumed that *C. tepidum* has two GGRs: one catalyzes phytyl formation in BChl *a* biosynthesis, and another catalyzes THGG formation in Chl *a* biosynthesis. However, Harada et al. constructed a *C. tepidum* mutant lacking the single *bchP* gene (CT2256) and showed that the mutant accumulated BChl *a* and Chl *a* both esterified with the GG group (17). This indicated that there is only one *bchP* gene responsible for GG reduction in the pigment biosynthesis of *C. tepidum* (17, 18). Harada et al. also made complementation experiments and introduced CT2256 into R. capsulatus strain lacking its authentic GGR. The R. capsulatus mutant was verified to produce phytylated BChl *a* (17). These indicated that GGR (gene product of CT2256) of *C. tepidum* exhibits potentially catalytic activities to reduce GG to phytyl moiety. By contrast, when it reacts with Chl *a*_GG_, the reduction of the C6=C7 double-bond of the isoprenoid tail is somehow inhibited or at least unachieved. In this study, we demonstrated that the GGR of *H. halochloris* itself is responsible for the THGG formation and that it has partial, unusual hydrogenation activities lacking the function of the C10=C11 double-bond reduction. Therefore, we conclude that the model proposed for *C. tepidum* GGR differs from that of *H. halochloris* GGR. In terms of catalyzing hydrogenation of the GG moiety only twice, the GGR of *H. halochloris* is likely to exhibit a novel catalytic mechanism and will provide insights into protein engineering. Additionally, the GGR variant, in which the N-terminal insertion peptides specific for *H. halochloris* were omitted (Hh_P_del), showed another-type partial activity catalyzing only a single hydrogenation reaction (Fig. 3, profile 5). The variant somehow acquired a novel reaction mode, which differs from the original GGR of *H. halochloris*.

**FIG 5:**
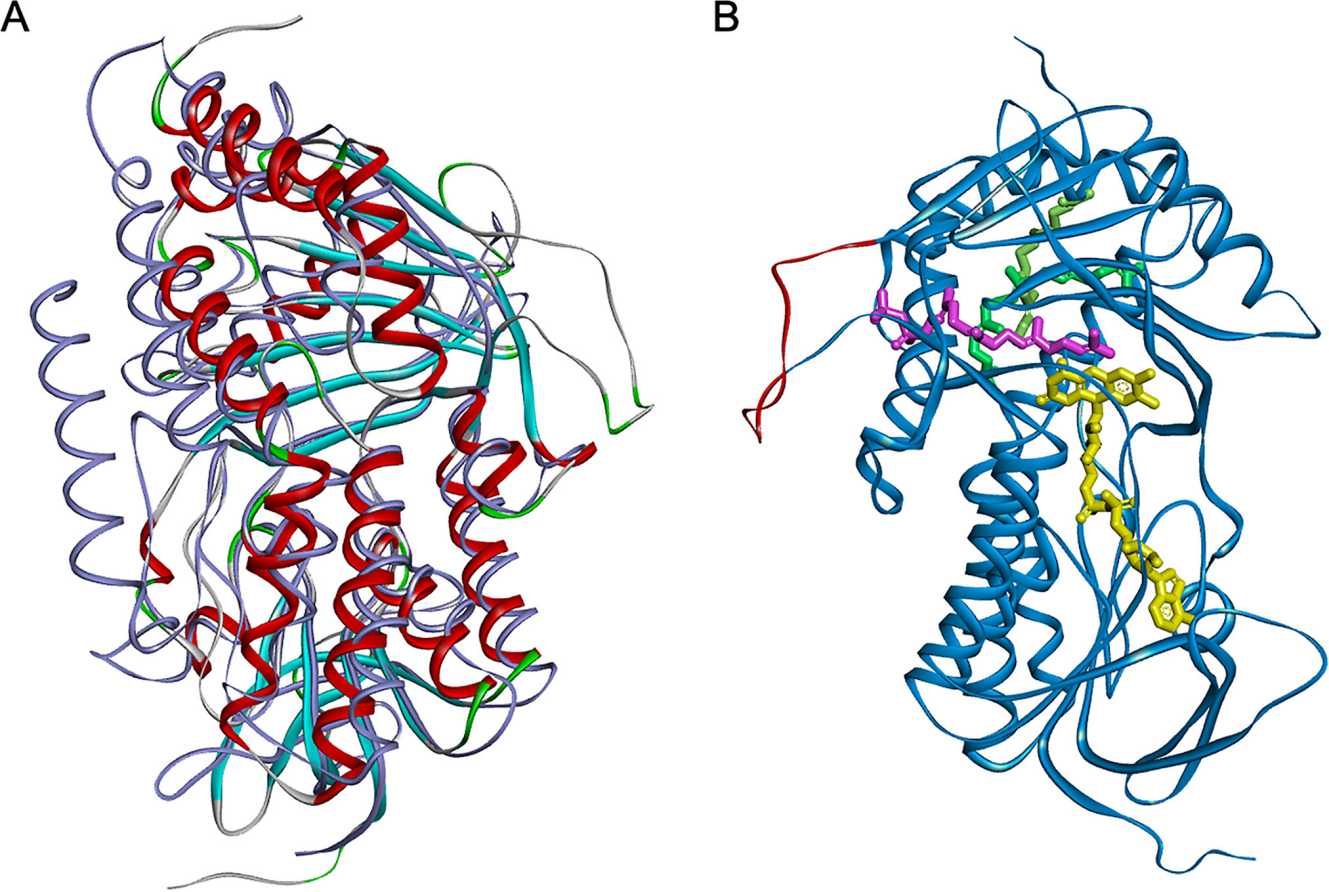
GGR structure prediction with AlphaFold. (A) The predicted structure of *H. halochloris* GGR (colored by secondary structure type) superimposed with the protein structure of S. acidocaldarius GGR (colored in gray, 4OPD, ref#22). (B) The predicted structure of *H. halochloris* GGR aligned with FAD (yellow) and three geranylgeranyl pyrophosphates (magenta, green, and light green). Positions of FAD and geranylgeranyl pyrophosphates were extracted from 4OPD after overlaying GGR structures of *H. halochloris* and S. acidocaldarius. The substrate geranylgeranyl pyrophosphate (colored in magenta), closest to FAD, locates in the vicinity of the looped insertion region characteristic for *H. halochloris* GGR (colored in red). Protein structure prediction was performed using the ColabFold platform (32), which is based on AlphaFold2 (33). The angle of view in B is horizontally rotated by roughly 180° from that in A.

Generally, GGR catalyzes the hydrogenation of carbon-carbon double bonds of unsaturated hydrocarbons to produce the corresponding single bond and works in various biosynthetic pathways for isoprenoid products, including α-tocopherols, phylloquinone, and archaeal cell membranes. One of the big enigmas on the catalytic mechanism of GGR is whether multiple hydrogenation reactions are successively conducted without releasing the substrate intermediates, or whether the intermediates (DHGG/THGG tails) dissociate from GGR before the next hydrogenation. The crystal structures of archaeal GGR from Sulfolobus acidocaldarius, including GGR bound to the substrate geranylgeranyl pyrophosphate, have been determined (22). In the 3D structure, the substrates were detected at three positions within GGR, although there is an active site in the vicinity of a single FAD. Therefore, it seems that the two observed substrates other than the one closest to the FAD were caught at binding pockets before or after hydrogenation reactions. In addition to site-directed mutation studies, the structure study of archaeal GGR has proposed the catalytic mechanism that the first and second hydrogenation might be processive and that the last third hydrogenation is probably not processive (22). The characteristic insertion of *H. halochloris* GGR (Fig. 4) is probably located close to the binding site that is relevant to the first and second hydrogenation in the archaeal GGR, according to the alignment of primary structures. Asparagine 90 and glycine 91 of S. acidocaldarius GGR (Fig. 4, colored in green) located in the vicinity of the pyrophosphate moiety of the substrate geranylgeranyl pyrophosphate (22) are at a similar position to the insertion region of *H. halochloris* GGR in the alignment (Fig. 4). We also performed protein structure prediction with AlphaFold for GGR of *H. halochloris* (Fig. 5). In the predicted structure, the characteristic insertion region constitutes a loop structure in the vicinity of the substrate binding site closest to FAD (Fig. 5B). These results could support the phenomenon that *H. halochloris* lacks hydrogenation at the C10=C11 position, which is probably the first hydrogenation event in other phototrophs, and that the variant GGR of Hh_P_del mutant lacks two hydrogenations, which are probably the first and second ones. However, we could not determine whether the DHGG moiety in the Hh_P_del mutant was a 2,6,10- or 2,10,14-phytatrienyl group. To determine the position of the unreduced double bond, a large number of pigment materials for analysis and the chemical standards of those intermediates are required. Further analysis on this will be reported elsewhere.

Using *Rhodobacter* species, *Rhodopseudomonas* species, barley, and radish sprouts, the order of the three hydrogenations by GGR was previously determined to be C10=C11, C6=C7, and then C14=15 (14) (see Fig. 1). Therefore, in the case of *H. halochloris*, the unachieved hydrogenation at the C10=C11 double bond corresponds to the first reaction (Fig. 1). In this study, the elution peaks of BChl *a*_DHGG_ and BChl *a*_THGG_ from mutants having *H. halochloris* GGRs were eluted slightly earlier than those prepared from *Rhodopseudomonas* sp. Rits strain (Fig. 3, profiles 3, 4, and 5). Mizoguchi et al. have reported HPLC elution profiles of BChl *b* esterified with two types of THGG moieties, namely, 2,10- and 2,14-phytadienyl tails, where BChl *b*_THGG_ with a 2,10-phytadienyl tail eluted slightly earlier than BChl *b*_THGG_ with a 2,14-phytadienyl tail (16). Similarly, BChl *b*_DHGG_ esterified with a 2,10,14-phytatrienyl tail eluted slightly earlier than BChl *b*_DHGG_ with a 2,6,14-phytatrienyl tail (16). These suggest that the isoprenoid moieties of BChl *a*_THGG_ and BChl *a*_DHGG_ from mutants with *H. halochloris* GGRs would be 2,10-phytadienyl and 2,10,14-phytatrienyl tails, respectively.

## MATERIALS AND METHODS

### Bacterial strains and culture conditions.

Rhodobacter sphaeroides J001 strain is a rifampicin-resistant derivative of R. sphaeroides 2.4.1 (23) used in this study for genetic manipulations. R. sphaeroides strains were cultivated in a PYS medium (24) at 30°C under oxic dark and anoxic light conditions. Furthermore, *Rhodopseudomonas* sp. strain Rits (21) was cultivated in a PYS medium at 30°C under anoxic light conditions. E. coli strains were cultivated in a Luria–Bertani (LB) medium at 37°C (25, 26). *H. halochloris* was grown in DSM253 medium at 42°C under anoxic light conditions, as instructed by Deutsche Sammlung von Mikroorganismen und Zellkulturen (DSMZ). Antibiotics were added to the media at the following concentrations: 25 μg/mL kanamycin, 100 μg/mL rifampicin, and 25 μg/mL streptomycin. Table 1 shows the strains and plasmids used in this study.

**TABLE 1:** Strains and plasmids used in this study^*a*^

Strain or plasmid	Relevant characteristics	Reference or source
E. coli		
DH5α	Host for cloning vectors	Toyobo Co.
JM109 λ-*pir*	Host of pJSC vector for cloning	25
S17-1 λ-*pir*	Host for pJSC derivatives to deliver to Δ*bchP* strain by conjugation	26
H. halochloris		
DSM 1059	Wild type	DSMZ
R. sphaeroides		
J001 (wild type)	Plating rifampicin resistance derivative from *Rba. sphaeroides* 2.4.1 (Rf^r^)	23
Δ*bchP*	*bchP* gene deleted mutant (Rf^r^, Km^r^)	This study
Hh_P_wt	Δ*bchP* introduced the pJ7-HhalbchP plasmid (Rf^r^, Km^r^, Sm^r^)	This study
Hh_P_del	Δ*bchP* introduced the pJ7-HhalPdel plasmid (Rf^r^, Km^r^, Sm^r^)	This study
Rs_P_wt	Δ*bchP* introduced the pJ7-RsphbchP plasmid (Rf^r^, Km^r^, Sm^r^)	This study
Rs_P_ins	Δ*bchP* introduced the pJ7-RsphPins plasmid (Rf^r^, Km^r^, Sm^r^)	This study
Plasmids		
pUCKM1	pUC plasmid with *neo* gene (Ap^r^, Km^r^)	27
pJSC	A derivative of suicide vector pJP5603 containing *sacB* and *cat* (Cm^r^)	29
pJN7	A derivative of broad-range host vector pBBR1MCS2 to which the *puc* promotor, BsaI restriction site, and Sm^r^ cartridge are embedded (Km^r^, Sm^r^)	9
pJSC-bchPKm	(pJSC+*bchP*::neo). Most of the *bchP* coding region of R. sphaeroides replaced with *neo* (Cm^r^, Km^r^)	This study
pJ7-HhalbchP	Intact *bchP* of *H. halochloris* cloned in pJN7 (Km^r^, Sm^r^)	This study
pJ7-HhalPdel	Variant *H. halochloris bchP* lacking the insertion region cloned in pJN7 (Km^r^, Sm^r^)	This study
pJ7-RsphbchP	Intact *bchP* of R. sphaeroides cloned in pJN7 (Km^r^, Sm^r^)	This study
pJ7-RsphPins	Variant R. sphaeroides *bchP* having the insertion region derived from *H. halochloris bchP*, cloned in pJN7 (Km^r^, Sm^r^)	This study

a Ap^r^, ampicillin resistance; Km^r^, kanamycin resistance; Rf^r^, rifampicin resistance; Sm^r^, streptomycin resistance; Cm^r^, chloramphenicol.

### Construction of R. sphaeroides mutants lacking GGR.

We amplified the upstream and downstream regions of the *bchP* gene in R. sphaeroides by PCR using two primer sets, bchPus-F and bchPus-R, and bchPds-F and bchPds-R, respectively (Fig. 2A, Table 2). The *neo* gene, which confers resistance to kanamycin, was amplified from the plasmid pUCKM1 (27) by PCR using the primer set, neo-F and neo-R (28). Using the In-Fusion HD Cloning Kit (TaKaRa-Bio, Shiga, Japan), we cloned the three amplified PCR products together into the SmaI site of the pJSC suicide vector (29) to obtain plasmid pJSC-bchPKm (Fig. 2A, Table 1).

**TABLE 2:** Sequences of primers for construction of the plasmids and confirmation of the mutants used in this study

Primer name	Primer sequence	Sequences for In-Fusion (underlined) overlapped with…
bchPus-F	TCGAGCTCGGTACCCTTCATGCAGGAGCTGATCCT	pJSC, SmaI site
bchPus-R	CGCTTCCTTTAGCAGACGAAGACATCATAGGCCAT	*neo* gene, 5′-end
bchPds-F	TGCTGGAGTTCTTCGCAAGATCGGGTTCAAGAACG	pJSC, SmaI site
bchPds-R	CTCTAGAGGATCCCCTGCAAGCCACAAGAAAAGGG	*neo* gene, 3′-end
neo-F	CTGCTAAAGGAAGCGGAACA	
neo-R	CGAAGAACTCCAGCATGAGA	
sphaP-comf-F	CTTCACCTTCTTCGTCTTCC	
sphaP-comf-F	GACCTCTTGCAAACGCAGAC	
Hhal-bchP-JF	CGAGAAGGGCGGCGCCCCAGTGGCCAATAATTCTGCTG	pJN7, BsaI site
Hhal-bchP-JR	CTGGGTACCGATATCTCAGCTCGAGCGCGCGAGG	pJN7, BsaI site
Hhal-bchP-middleR	GATATCCATGTCAGCATGG	Hhal-bchP-middleAF
Hhal-bchP-middleAF	GCTGACATGGATATCCCCTTGAGCATGCTGAGCATG	Hhal-bchP-middleR
Rsph-bchP-JF	CGAGAAGGGCGGCGCCGCCTATGATGTCTTCGTAGTG	pJN7, BsaI site
Rsph-bchP-JR	CTGGGTACCGATATCTCAGGTCCATTGCGGCGAG	pJN7, BsaI site
Rsph-bchP-insertR^*a*^	*CTGGCGGAAGGGCCACTCCGGGGGCGGG*GATCGGG ATGTCGACCTTGC	Rsph-bchP-insertAF
Rsph-bchP-insertAF	*TGGCCCTTCCGCCAGACGCCAAAGACGGG*GAGGGC GGCTTCGTCGG	Rsph-bchP-insertR

a Italicized sequences in Rsph-bchP-insertR and Rsph-bchP-insertAF correspond to the coding region for the inserted peptides.

The plasmid pJSC-bchPKm was transferred into the wild-type strain R. sphaeroides using a conjugation method with E. coli strain S17-1 λ-*pir* (26, 29). We selected the kanamycin-resistant colonies grown in the presence of 5% sucrose as double-crossover candidates. Then, we performed analytical PCR to verify the chromosomal insertion of the *neo* gene into the *bchP* locus of genomic DNA (Fig. 2A). Using sphaP-comf-F and sphaP-comf-R primers (Table 2), the size of the PCR product from the wild-type strain of R. sphaeroides is expected to be 2.48 kbp, which was observed in Fig. 2B, lane 1. The same primer set amplified an approximately 0.18 bp-longer fragment for the mutant strain (Fig. 2B, lane 2). These results indicate that the kanamycin-resistance gene was introduced into the targeted *bchP* gene in the genome of the mutant, which is hereafter called Δ*bchP* mutant.

### Complementation mutants carrying wild-type and variant GGR.

To construct the mutant complemented with *H. halochloris* GGR, we amplified the *bchP* gene of *H. halochloris* by PCR using primers, Hhal-bchP-JF and Hhal-bchP-JR (Table 2). Using the In-Fusion technique, we cloned the DNA fragment into the BsaI site of the streptomycin-resistant plasmid vector pJN7 (9) to yield pJ7-HhalbchP. The plasmid was transformed into Δ*bchP* mutant by conjugation with the E. coli strain S17-1 (29). Transconjugant colonies were selected on PYS plates containing streptomycin (25 µg/mL), kanamycin (25 µg/mL), and rifampicin (100 µg/mL). Colonies were selected from the third round of selective plates and cultivated in a liquid PYS medium. To verify that conjugation was successfully achieved, plasmids were extracted from the liquid cultures and confirmed to be pJ7-HhalbchP by cutting with the appropriate restriction enzymes and amplifying the *bchP* gene. This mutant was designated Hh_P_wt. Note that the introduction of plasmid pJ7-HhalbchP provided the heterologous expression of *H. halochloris bchP* under the transcriptional control of the *puc* operon promoter derived from R. sphaeroides.

The mutant complemented with a variant GGR of *H. halochloris*, in which the characteristic insertion region at the N-terminus was deleted, was constructed as follows. DNA fragments containing partial *bchP* gene regions of *H. halochloris* were amplified by PCR using two sets of primers, Hhal-bchP-JF and Hhal-bchP-middleR, and Hhal-bchP-middleAF and Hhal-bchP-JR (Table 2). The two PCR fragments were cloned together into the BsaI site of plasmid pJN7, yielding plasmid pJ7-HhalPdel. Then, we transferred the plasmid into the Δ*bchP* mutant by conjugation as described above, and the obtained complementation mutant was designated Hh_P_del.

For the control mutant strain complemented with wild-type R. sphaeroides GGR, the *bchP* gene of R. sphaeroides was amplified by PCR using primers, Rsph-bchP-JF and Rsph-bchP-JR (Table 2). The PCR fragment was cloned into the BsaI site of pJN7 using the In-Fusion method, yielding pJ7-RsphbchP. Then, the plasmid was transferred into the Δ*bchP* mutant by conjugation as described above. The resultant complementation mutant was designated as Rs_P_wt. Also, the complementation mutant with a variant GGR of R. sphaeroides was constructed similarly. Additional peptides from the characteristic insertion region of *H. halochloris* GGR was embedded in the variant GGR of R. sphaeroides in the mutant. Two DNA fragments containing *bchP* of R. sphaeroides front and back from the insertion region were amplified by PCR using two sets of primers: Rsph-bchP-JF and Rsph-bchP-insertR; and Rsph-bchP-insertAF and Rsph-bchP-JR (Table 2). The primers, Rsph-bchP-insertR and Rsph-bchP-insertAF, include sequences corresponding to the coding region for the inserted peptides, PAPGVALPPDAKDG (Table 2). The two fragments were cloned together into the BsaI site of plasmid pJN7 using the In-Fusion method, yielding the plasmid pJ7-RsphPins, and the following conjugation, selection, and genetic verification were conducted as for other complementation mutants. The resultant mutant was designated Rs_P_ins.

### HPLC analysis.

The wild-type and mutant strains were cultivated in a PYS medium under semi-oxic dark and anoxic light conditions for pigment analysis. Note that *Rhodobacter* species typically accumulate BChl pigments even under dark conditions. The HPLC results depicted in the figure were obtained using pigments extracted from cells grown under dark conditions; however, HPLC analysis using pigments from cells grown under light conditions yielded the same results. We harvested the cells using centrifugation, extracted the pigments with acetone/methanol (7:2, vol/vol), and filtered them with a PVDF membrane. We performed reverse-phase HPLC measurements using an octadecylated silica gel column (Cosmosil 5C_18_-AR-II 4.6 mmϕ × 250 mm, 5 μm, Nacalai Tesque, Kyoto, Japan) with a mobile phase, methanol:acetone:water = 82:15:3 with a flow rate of 0.4 mL/min.
